# Wavelength-Specific Artificial Light Disrupts Calling Behavior, Pheromone Blend Composition, and Mating Success in the Fall Armyworm, *Spodoptera frugiperda* (Lepidoptera: Noctuidae)

**DOI:** 10.3390/insects17080809

**Published:** 2026-08-04

**Authors:** Fida Hussain Magsi, Amees Ullah, Yihui Ma, Jianrong Huang, Guoping Li, Xincheng Zhao, Hongqiang Feng

**Affiliations:** 1 Henan Key Laboratory of Agricultural Pest Monitoring and Control, No. 0 Entomological Radar Field Scientific Observation and Research Station of Henan Province, Key Laboratory of Integrated Crop Pests Management on Crops in Southern Region of North China of Ministry of Agriculture and Rural Affairs, Institute of Plant Protection, Henan Academy of Agricultural Sciences, Zhengzhou 450002, China; fidajanmagsi@yahoo.com (F.H.M.);; 2 College of Plant Protection, Henan Agricultural University, Zhengzhou 450046, China

**Keywords:** LED illumination, chemical ecology, pheromone blend, reproductive communication, integrated pest management

## Abstract

Artificial light at night (ALAN) and the broader use of artificial light in agriculture are increasingly recognized to affect the physiology and behavior of nocturnal insects, yet their influence on pheromone-mediated reproductive communication in economically important insect pest species remains poorly understood. Here, we investigated the effects of artificial illumination on calling behavior, sex pheromone production, blend composition, and mating success in the fall armyworm *Spodoptera frugiperda*, under both laboratory and semi-field conditions. Virgin females were exposed during the scotophase to one of four wavelength-specific treatments, including dark control, red (620 nm), green (520 nm), or blue (460 nm) LED light. Female calling behavior occurred during the early-to-mid scotophase under dark and red light conditions, while it was significantly delayed and suppressed under blue and green light. Specifically, blue light suppressed production of the key pheromone component (*Z*)-9-tetradecenyl acetate (*Z*9-14:Ac) and enhanced (*Z*)-11-hexadecenyl acetate (*Z*11-16:Ac), altering the species-specific pheromone blend composition. Mating success was significantly reduced under blue light in both laboratory and semi-field experiments. Under controlled laboratory conditions, green light also affected mating success, but this effect was not significant in the semi-field experiment. Blue light imposed the strongest inhibitory effect, reducing mating success by 39% relative to the dark condition. Red light produced minimal effects in all measured parameters, consistent with reduced sensitivity to longer wavelengths in nocturnal moths. These results demonstrate that wavelength-specific artificial light can disrupt several components of the reproductive communication system in *S. frugiperda* and suggest that manipulation of artificial light may offer a non-chemical approach for disrupting mating behavior in economically important insect pest species within integrated pest management programs.

## Simple Summary

The fall armyworm (*Spodoptera frugiperda*) is one of the world’s most devastating insect pests. Like many moths, it relies on sex pheromones to find a mate. Because artificial lighting is increasingly common around farmland, we tested whether different colors of light disrupt this process. In laboratory and field cage experiments, among the tested lights, blue light had the strongest effect on female calling behavior and pheromone blend composition, and it reduced successful matings. These findings suggest that carefully chosen light could serve as a potential strategy to manage fall armyworm.

## 1. Introduction

The fall armyworm, *Spodoptera frugiperda* (J.E. Smith, 1797) (Lepidoptera: Noctuidae), is native to tropical and subtropical regions of the Americas and has emerged as one of the most economically damaging invasive pests globally. Since its detection in sub-Saharan Africa, the species has expanded its range across Asia, Australia, and most recently the Middle East, establishing in diverse agroecosystems and causing significant yield losses [1,2,3]. The pest possesses a broad host range, high reproduction, long-distance migratory potential, and adaptability to varying environmental conditions [3]. Effective management of *S. frugiperda* therefore requires a detailed understanding of its biology and behavior, including the environmental factors and their influence on reproduction and population dynamics. Artificial light at night (ALAN) is a growing environmental factor that disrupts physiological and behavioral activities of nocturnal insects [4,5]. Insects possess well-developed photoreceptive systems that enable them to detect light intensity and spectral composition, which play a key role in biological processes including orientation, development, circadian rhythm, and reproduction [4,6,7]. The composition of ALAN is particularly important because different wavelengths vary in their biological impacts, and nocturnal insects exhibit significant sensitivity to shorter wavelengths, particularly the blue and ultraviolet spectrum, compared to longer wavelengths [8,9]. Similarly, recent reports have demonstrated minimal activity of nocturnal moths under enhanced lighting [10,11]. Beyond its agricultural importance, ALAN is also recognized as a driver of insect decline in conservation research, where nocturnal moths are among the taxa most affected by nighttime lighting through impaired flight, foraging, and reproduction [12,13]. Apart from disrupting activity patterns, ALAN has been demonstrated to affect pheromone-based chemical communication, mating behavior, and reproduction activities in diverse insect taxa [14,15].

Beyond agricultural contexts, a growing body of conservation-oriented research has documented the impacts of ALAN on moth populations, including reduced flight-to-light and nocturnal activity, disrupted reproduction, and contributions to widespread moth declines [16], underscoring that the wavelength sensitivities examined here are relevant to both pest management and the conservation of non-target nocturnal Lepidoptera. In moths, reproduction relies mainly on pheromone-mediated chemical communication. Calling females release species-specific sex pheromone compounds during the period of the scotophase, regulated by the circadian clock and controlled by neuropeptide signaling, specifically, pheromone biosynthesis activating neuropeptide (PBAN) [17,18,19]. The male moth detects these female-released cues using well-evolved olfaction, where successful mate location depends not only on major pheromone compounds but also on their specific ratios; even minor changes in blend composition can hinder mate recognition and reduce attraction [20,21,22,23]. Pheromone communication is highly vulnerable to environmental disruption; previous research has revealed that ALAN can influence behavior, reduce pheromone quantities, and modify pheromone blends in moth species [24,25]. In addition, ALAN can affect reproductive outcomes by inhibiting synchrony between male and female responsiveness [26,27]. It is important to distinguish, however, between the low intensity characteristic of ambient light pollution and the far higher, close-range intensities produced by artificial light sources used in applied settings such as pest monitoring and control, where nocturnal moths are routinely exposed to bright illumination. In the context of pest monitoring, use of artificial illumination, specifically, blue LED for *S. frugiperda*, elicits strong positive phototaxis [28,29]. Recent reports demonstrated negative effects of blue and green LED on survival, development, and fecundity across the developmental stages of *S. frugiperda*, particularly under short-wavelength illumination [30]. Furthermore, the influence of light pollution during migration has also been reported, where *S. frugiperda* is selecting darker sky regions and avoiding light-polluted habitats during flight [31]. However, despite this evidence, the effects of wavelength-specific artificial light on the reproductive communication of *S. frugiperda*, particularly calling behavior, pheromone production, pheromone blend composition, and mating success, have not been investigated. This is a crucial gap, because disruption of pheromone chemical communication has direct implications for reproductive success and may offer sustainable pest management strategies. Therefore, the present study was designed to systematically investigate the effects of artificial illumination on the reproductive behavior and chemical communication of *S. frugiperda* under laboratory and semi-field conditions. We aimed to determine the effects of blue, green, and red artificial lights on temporal patterns of calling behavior, quantification of sex pheromone production, light-induced variation in pheromone blend composition, and the influence of each light on mating success.

## 2. Material and Methods

### 2.1. Chemicals

The synthetic sex pheromone compounds (*Z*)-7-dodecenyl acetate (*Z*7-12:Ac, ≥97% purity) and (*Z*)-9-tetradecenyl acetate (*Z*9-14:Ac, ≥97% purity) were purchased from Macklin Co., Ltd. (Shanghai, China), and (*Z*)-11-hexadecenyl acetate (*Z*11-16:Ac, ≥97% purity) was obtained from Leyan Chemicals Co., Ltd. (Shanghai, China). n-Hexane (HPLC grade) and heptadecane (internal standard, ≥99% purity) were obtained from Sigma-Aldrich (Shanghai, China).

### 2.2. Insects

Larvae of *S. frugiperda* were collected in late August 2025 from a maize field at the Xinxiang experimental base of the Chinese Academy of Agricultural Sciences, Henan Province, China (113.77° E, 35.13° N). To maintain genetic variability, field-collected larvae were incorporated into the insect colony at intervals of approximately three to four generations. Larvae were reared on a maize-leaf- and wheat-bran-based artificial diet [32], under controlled laboratory conditions (25 ± 1 °C, 70 ± 5% RH, 15L:9D photoperiod). Larvae were reared in transparent plastic tubes (length 95.1 mm and diameter 23.1 mm), and the openings of the plastic tubes were covered with standard white sponge (24 × 24 mm). One larva was placed per container to prevent cannibalism. After pupation, males and females were separated and maintained in mesh cages (55 × 55 × 55 cm, length × width × height) until eclosion (50 insects/cage). Adults were supplied with 10% honey solution. Only healthy, virgin adults aged 2–3 days post-eclosion were used in all experiments. Individuals used in one experiment were not reused in subsequent experiments.

### 2.3. Calling Behavior

The calling behavior of virgin females (2–3 days old) was evaluated under four different light conditions during the scotophase. Females were placed separately in transparent plastic containers (8.1 cm upper diameter, 4.6 cm base diameter, and 10.5 cm height) sealed with thin transparent polypropylene film. Containers were placed in the experimental room at 25 ± 1 °C, 70 ± 5% RH under a 15L:9D photoperiod. During the photophase, insects were exposed to normal white fluorescent illumination. At the onset of scotophase, insects were assigned to one of four treatments: (i) dark control, (ii) blue LED (460 ± 5 nm, 50 W, 1721 lux), (iii) green LED (520 ± 5 nm, 50 W, 1746 lux), and (iv) red LED (620 ± 5 nm, 50 W, 1092 lux). All LED sources were supplied by Zhongshan Yichen Co., Ltd. Zhongshan, Guangdong, China. All light illumination treatments were positioned at 120 cm above the insects to ensure uniform illumination across the experimental area. Light intensity was measured using a digital light meter (model ZTW1702A, range 0.1–200,000 lux, Zhejiang Zhengtai Technology Co., Ltd., Wenzhou, China) prior to observations. Light intensity in all treatments was characterized photometrically as illuminance (lux); illumination was recorded as horizontal illuminance at the position occupied by the moths (120 cm below the light source) using a digital light meter (ZTW1702A). During the 15 h photophase, insects were exposed to white fluorescent illumination measured at 2430 lux at the level of the insects. The three LED colors are reported by their nominal peak wavelengths as specified by the manufacturer: blue (peak 460 nm), green (peak 520 nm), and red (peak 620 nm). Insects were continuously exposed to assigned treatments throughout the entire scotophase. Calling activity was recorded at hourly intervals. During dark-control observations, a dim red light was used for a 30 s observation window and was turned off immediately. At each time point, females displaying the characteristic calling posture as described by [33] and abdominal elevation were defined as displaying calling activity. For each light treatment, three independent replicates were conducted, each using a separate group of 20 virgin females (60 females evaluated in total per treatment). Within each replicate, the calling behavior of all females was monitored throughout the scotophase.

### 2.4. Pheromone Extraction and Gas Chromatography–Mass Spectrometry (GC–MS) Analysis

For pheromone extraction, calling females were removed from the containers and glands were extruded by gentle abdominal pressure and excised with fine forceps. Glands were immersed in 100 μL n-hexane, and 5 μL of heptadecane internal standard solution (1 μg/mL in n-hexane) was added to each sample. Each sample contained the glands of either 10 or 5 females to ensure detectable quantities of minor pheromone components, and each pooled sample constituted one biological replicate. GC–MS analysis was performed using an Agilent 7890B gas chromatograph coupled to an Agilent MSD 5977B mass spectrometer (Agilent Technologies, Folsom, CA, USA), equipped with a DB-23 polar column (30 m length × 0.25 mm inner diameter, 0.25 μm film thickness; J&W Scientific, Agilent Technologies, Folsom, CA, USA). Samples (1 μL) were injected in splitless mode at an inlet temperature of 200 °C, with helium as the carrier gas at a constant flow rate of 1 mL/min. The oven temperature program was as follows: 50 °C initial, held 2 min; ramped at 10 °C/min to 160 °C, then ramped at 4 °C/min to 220 °C, and held 10 min. The mass spectrometer was operated in electron impact (EI) ionization mode at 70 eV, scanning *m*/*z* 40–500. All samples were stored at −20 °C until analysis. Pheromone components were identified by comparison of both mass spectra and retention times with those authentic synthetic standards analyzed under identical GC–MS conditions. Compound quantities were calculated from the internal standard response and expressed as ng per female.

### 2.5. Mating Success Under Laboratory Conditions

The effect of artificial illumination on mating success was evaluated under controlled laboratory conditions (25 ± 1 °C, 70 ± 5% RH, 15L:9D photoperiod). Virgin males and females (2–3 days old) were paired in transparent plastic containers and introduced to experimental room. Each pair of male and female was placed in a plastic container (upper diameter 8.1 cm, lower diameter 4.6 cm and height 10.5 cm). A total of 30 independent insect pairs were tested for each light treatment. Each pair was supplied with 10% honey solution. Mating success was assessed by visual observation of copulation. Following the experiment, mated females were transferred individually to plastic containers and placed under standard conditions (25 ± 1 °C, 70 ± 5% RH, 15L:9D photoperiod). Oviposited eggs were monitored for hatching, and successful larval eclosion was used to confirm egg fertility and validate mating success.

### 2.6. Pheromone Production Under Light Conditions

The effect of ALAN on sex pheromone production was evaluated using 2–3-day-old virgin females. Females were individually exposed to the same four light treatments described in above section, with LED sources positioned 120 cm above the insects. Females were exposed to light illumination throughout the scotophase period. Pheromone gland extraction was performed at the peak calling time point for each treatment, as determined from the calling behavior experiment (hour 6 for dark and red light, 7 for blue and 8 for green light). For each replicate, 10 individual females were used, and the experiment was repeated with 3 replicates. Glands from calling individuals were extracted and analyzed by GC–MS.

### 2.7. Temporal Dynamics of Sex Pheromone Production

To investigate how light quality influences the temporal profile of pheromone production, virgin females 2–3 days old were exposed to light environments, the same as described in previous sections. Pheromone glands were extracted from five females per treatment at four time points (2, 4, 6, and 8 h) during the scotophase. The gland extraction method was similar to that mentioned in Section 2.4. For each replicate, 5 individual females were used, and the experiment was repeated with 3 biological replicates. Mean pheromone quantities (±SE) were calculated for each compound, and the effects of light treatment, time and their interaction were assessed.

### 2.8. Influence of Light on Mating Status of Fall Armyworm Under Semi-Field Conditions

**Figure 1:**
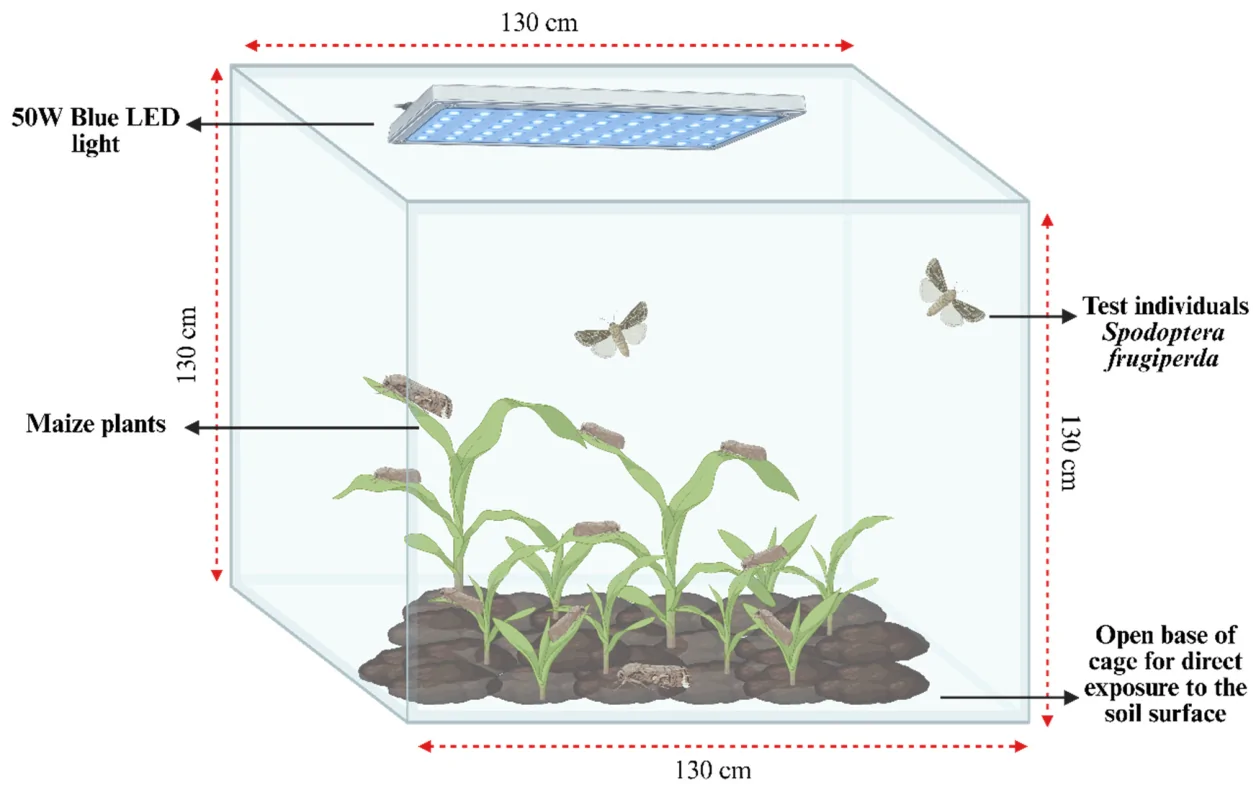
Schematic illustration of semi-field experiment to evaluate the influence of artificial light on mating status of *Spodoptera frugiperda*.

The effect of artificial illumination on mating success was further evaluated under semi-field conditions, in a maize plantation at the Xinxiang Experimental Base, Henan Province, China (113.77° E, 35.13° N). Square muslin cloth cages (130 cm × 130 cm × 130 cm length, width, and height) were placed within the maize crop, with the base left open to allow contact with the soil surface. The same LED sources used in the laboratory were used in the semi-field experiment and illuminance was measured at the level of the ground within each cage (blue 1440 lux, green 1370 lux, and red 1005 lux). The lights were suspended centrally from the upper panel of each cage (Figure 1) via a fine wire passed through a small aperture in the fabric. Light treatments were activated at dusk and deactivated at dawn each day. For each replicate, 10 virgin females, 2–3 days old, were introduced into the cage at the onset of the scotophase. After two hours of light exposure (to allow females to acclimatize to the natural environment), 10 virgin males of the same age were introduced into the cage. Adults were supplied with a 10% honey solution. Each treatment was replicated five times on independent experimental nights. The following morning, all insects were recaptured. Females were individually transferred to plastic oviposition containers under standard laboratory conditions (25 ± 1 °C, 70 ± 5% RH, 15L:9D photoperiod) and monitored for oviposition. A female was considered successfully mated if eggs hatched, indicating successful fertilization. The experiments were conducted in the experimental field of maize, where no pesticides were applied before or after experiment. The mean temperature during experimental nights was (18 ± 3 °C) based on meteorological data from the China Meteorological Administration.

### 2.9. Statistical Analysis

The effects of light treatment, time, and their interaction on female calling behavior and temporal pheromone production were analyzed by two-way analysis of variance (ANOVA), followed by Tukey’s HSD post-hoc test. The production of each pheromone compound (*Z*7-12:Ac, *Z*9-14:Ac, and *Z*11-16:Ac) across light treatments was analyzed separately by one-way ANOVA followed by Tukey’s HSD. Mating success data were analyzed using a generalized linear model (GLM) with binomial error distribution, and significance effect was assessed using a Wald chi-square test, followed by Bonferroni-corrected pairwise comparisons. All results are reported as mean ± standard error (SE), and statistical significance was set at *p* < 0.05. All statistical analyses were performed using SPSS Statistics 21.0 (IBM, Armon, NY, USA).

## 3. Results

### 3.1. Effect of Light Treatments on S. frugiperda Calling Behavior

**Figure 2:**
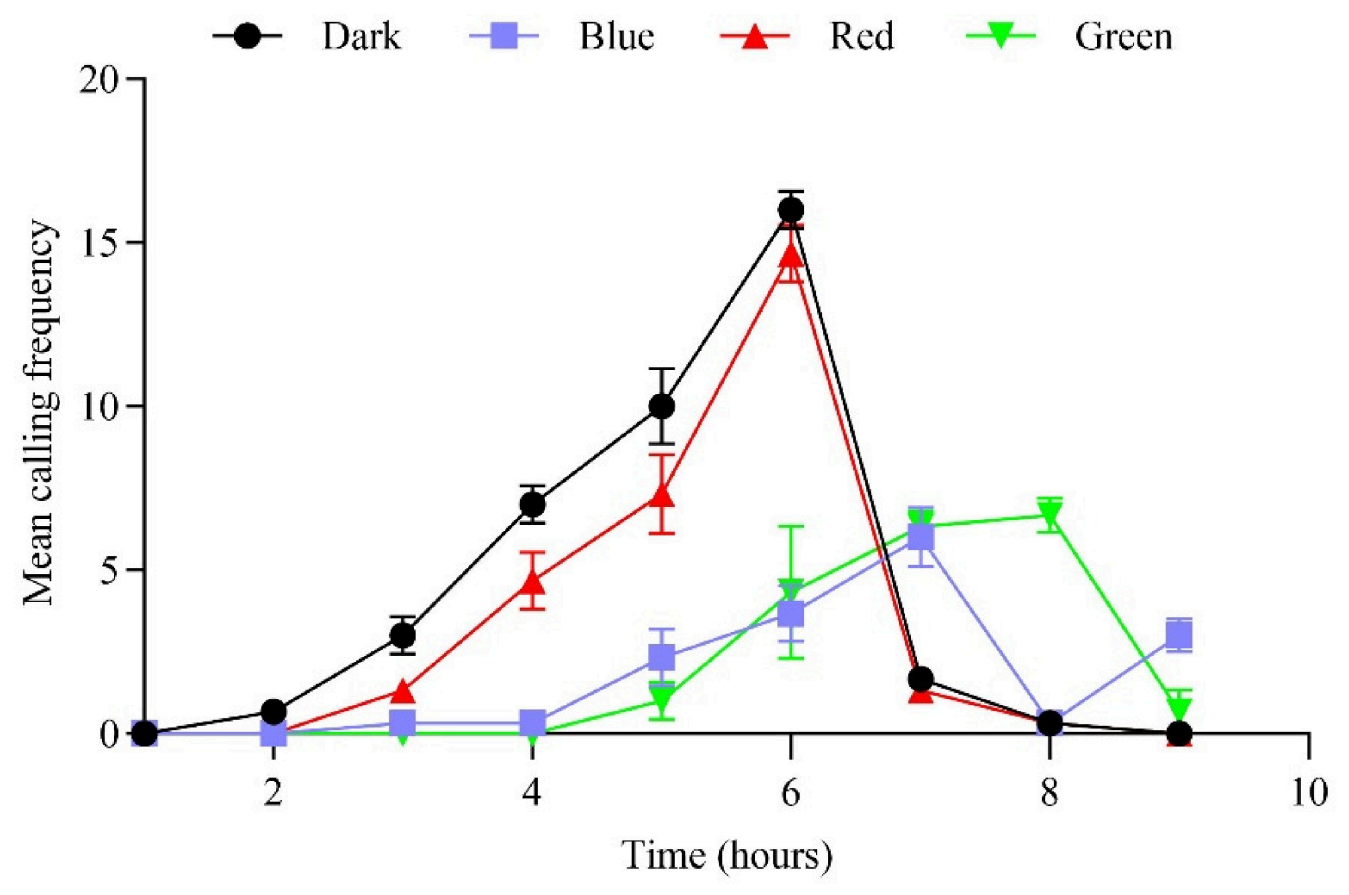
Temporal variation in *Spodoptera frugiperda* female calling behavior under different light treatments. Values represent mean ± SE (*n* = 3, independent observations).

The calling behavior of *S. frugiperda* was significantly affected by light treatment during the scotophase, and results revealed significant influences of color of illumination (F_(3,72)_ = 7.778, *p* < 0.001), time (F_(8,72)_ = 24.588, *p* < 0.001), and their interaction (F_(24,72)_ = 6.665, *p* < 0.001) (Figure 2) on calling frequency. Calling behavior started early in the scotophase and peaked at hour 6 under dark and red light conditions, followed by a rapid decline. Blue and green light delayed and suppressed calling activity; under blue light, the calling peak shifted to hour 7, while under green light, calling was negligible until hour 5 and peaked at hour 8. Overall, short-wavelength illumination postponed the peaking time of calling behavior and suppressed the frequency of calling as compared with red and dark control conditions.

### 3.2. Gas Chromatography and Mass Spectrometry (GC–MS) Analysis

**Figure 3:**
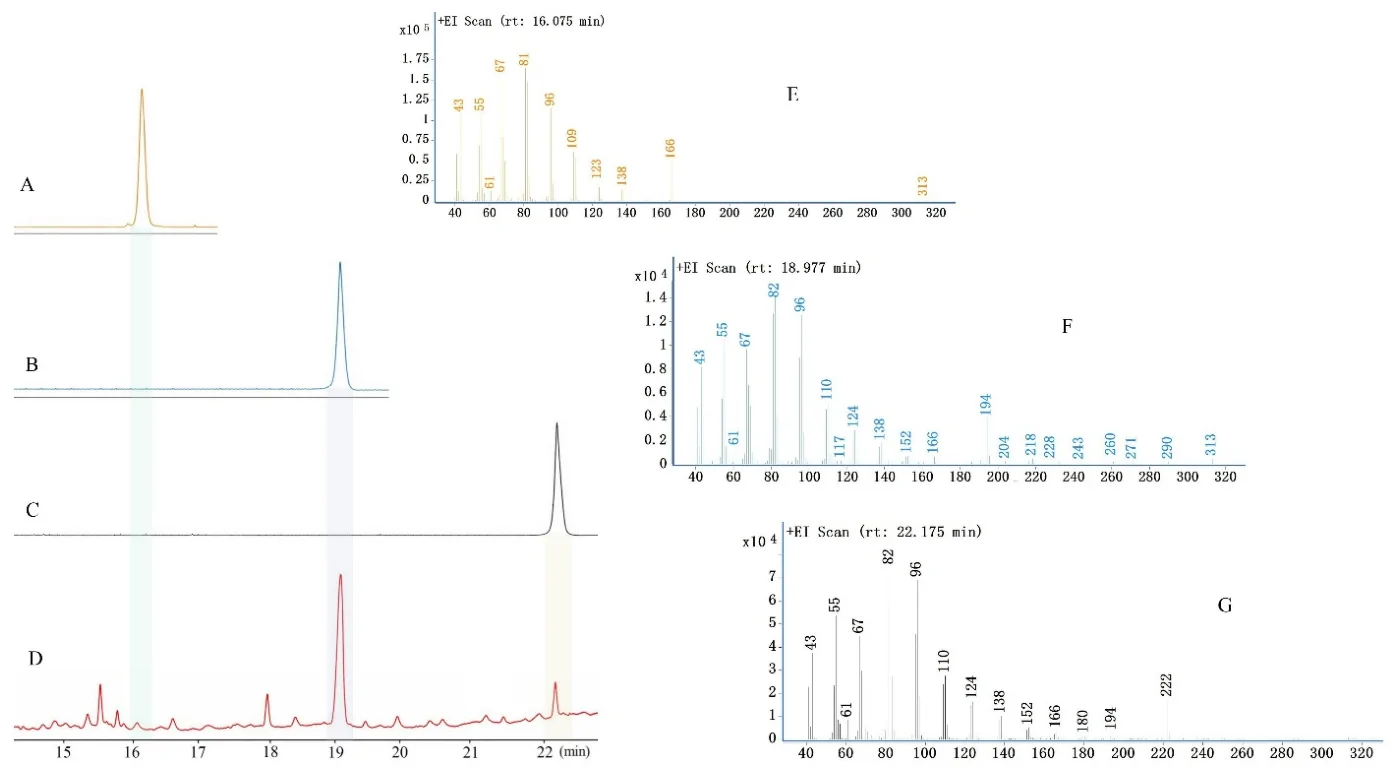
Gas chromatography and mass spectrometry analysis of *Spodoptera frugiperda* pheromone gland extracts: the total ion chromatographs of female gland extracts and standards, (**A**) synthetic *Z*7-12:Ac, (**B**) synthetic *Z*9-14:Ac, (**C**) synthetic *Z*11-16:Ac, (**D**) female pheromone gland extracts, (**E**) mass spectra of *Z*7-12:Ac, (**F**) mass spectra of *Z*9-14:Ac, and (**G**) mass spectra of *Z*11-16:Ac.

Gas chromatography–mass spectrometry (GC–MS) analysis of extracts from female pheromone glands of *S. frugiperda* revealed the presence of three components in the total ion chromatogram (TIC) (Figure 3). Pheromone compound (*Z*)-7-dodecenyl acetate (*Z*7-12:Ac) eluted at a retention time of 16.07 min, (*Z*)-9-tetradecenyl acetate (*Z*9-14:Ac) at 18.97 min, followed by the third compound, (*Z*)-11-hexadecenyl acetate (*Z*11-16:Ac), which eluted at 22.18 min. Identification of compounds was confirmed by comparing the retention time and spectral information with synthetic standards.

### 3.3. Mating Status Under Different Light Conditions in Laboratory

**Figure 4:**
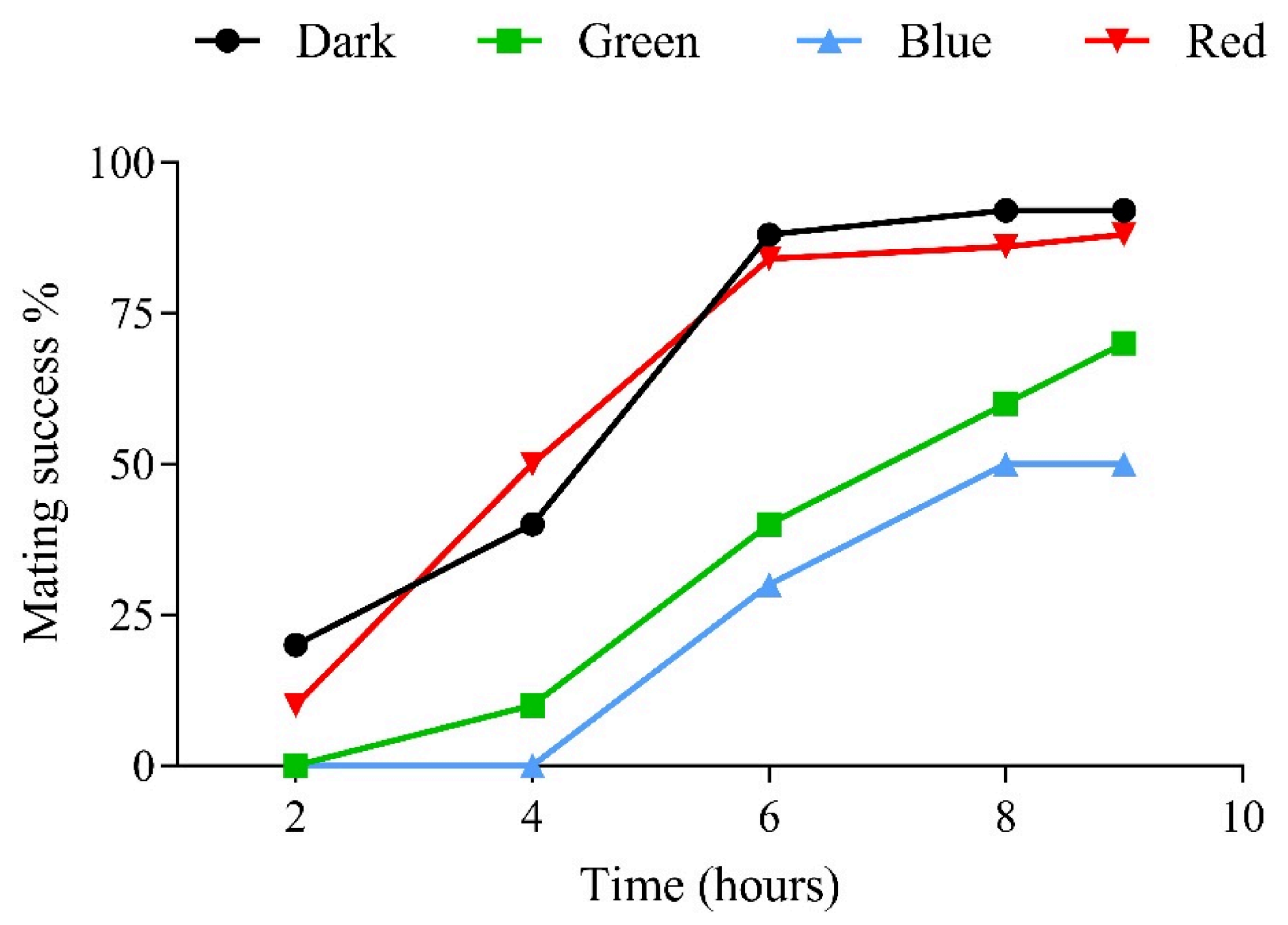
Effect of light treatments on mating success of *Spodoptera frugiperda* over time. Values represent the percentage of mated pairs under dark, green, blue, and red light conditions. Statistical analysis was performed using generalized linear model with binomial error distribution (*p* < 0.05).

Light treatments highly influenced the mating status of *S. frugiperda* over time and revealed significant difference among the treatments (χ^2^ = 125.697, df = 3, *p* < 0.001) (Figure 4), indicating that light treatment and time remarkably influenced mating success. The higher mating success rate was observed in the control group, which was followed by the red light, green light, and blue light. Experimental results further showed that blue lights highly influenced the mating status and there were no mated pairs found at early hours of the scotophase; mating was first recorded at hour 4 of the scotophase and increased gradually in blue light. Overall, these results demonstrate that mating success in *S. frugiperda* is strongly influenced by color of illumination, with dark environments promoting mating, while blue light significantly reduced the mating success.

### 3.4. Pheromone Production

**Figure 5:**
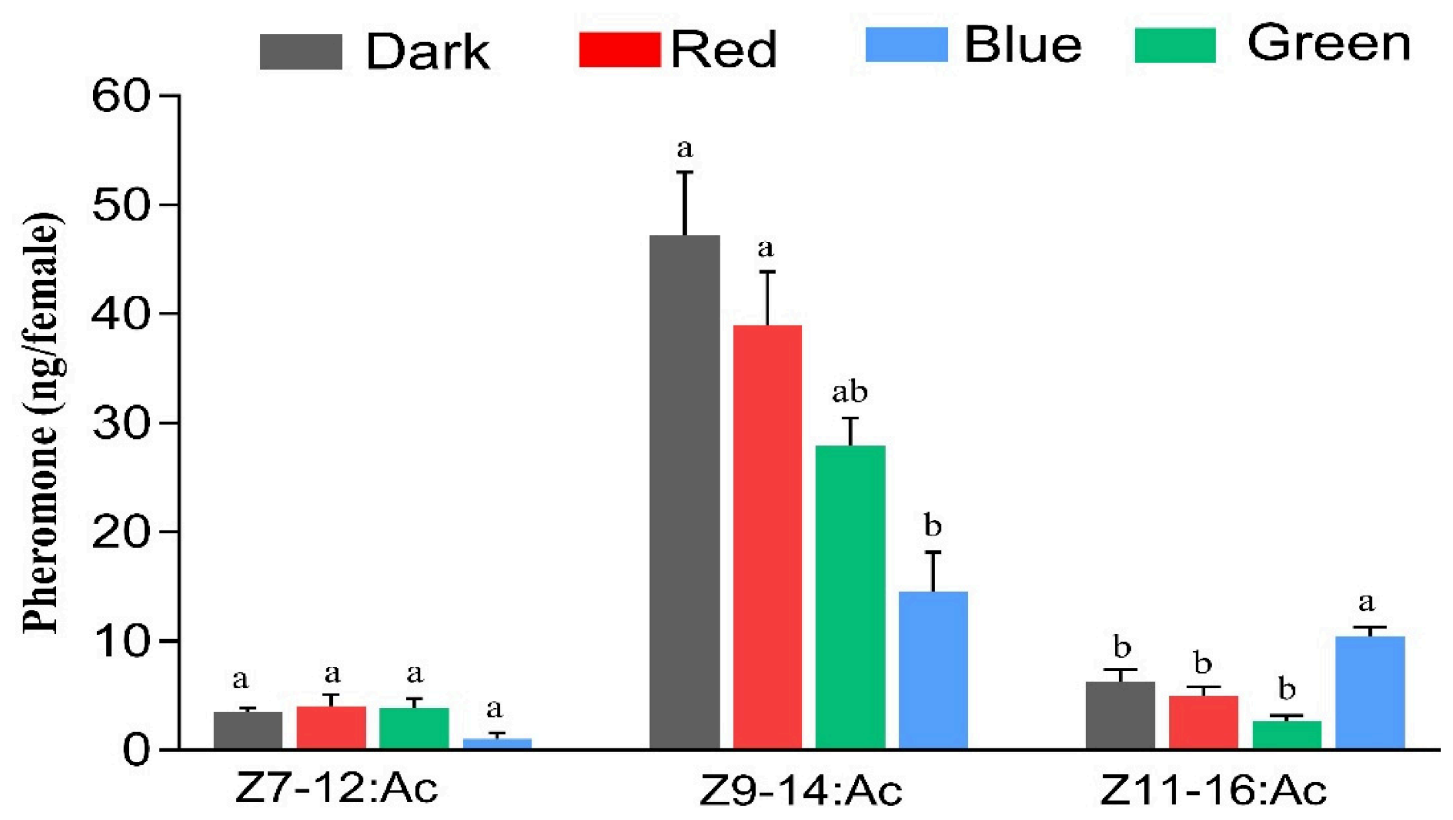
Effect of artificial light on sex pheromone production in *Spodoptera frugiperda*. Mean (± SE) production (ng/female) of *Z*7-12:Ac, *Z*9-14:Ac, and *Z*11-16:Ac extracted from pheromone glands (*n* = 3, replicates per treatment) under dark, red, green, and blue light conditions. Different lowercase letters indicate significant differences among treatments within each compound (one-way ANOVA, followed by Tukey’s HSD, *p* < 0.05).

Light treatment significantly affected the amount of *Z*9-14:Ac (F_(3,8)_ = 10.478, *p* = 0.004) and *Z*11-16:Ac (F_(3,8)_ = 15.685, *p* = 0.001) produced by female *S*. *frugiperda*, but had no significant effect on *Z*7-12:Ac production (F_(3,8)_ = 3.522, *p* = 0.069) (Figure 5). *Z*9-14:Ac remained the predominant pheromone component under all light conditions. However, its production was significantly lower under blue light than dark and red light, while no significant difference was observed between the dark and red treatments (Tukey’s HSD, *p* > 0.05). Although *Z*7-12:Ac tended to be lower under blue light, this trend was not statistically significant, and all four treatments fell within a single homogeneous subset according to Tukey’s HSD (*p* = 0.081). In contrast, *Z*11-16:Ac production was significantly enhanced under blue light compared with all other treatments (Tukey’s HSD, *p* ≤ 0.032). Overall, these findings indicate that blue light altered the pheromone blend composition of *S. frugiperda* by reducing the major pheromone component *Z*9-14:Ac while enhancing *Z*11-16:Ac.

### 3.5. Temporal Pheromone Dynamics

**Figure 6:**
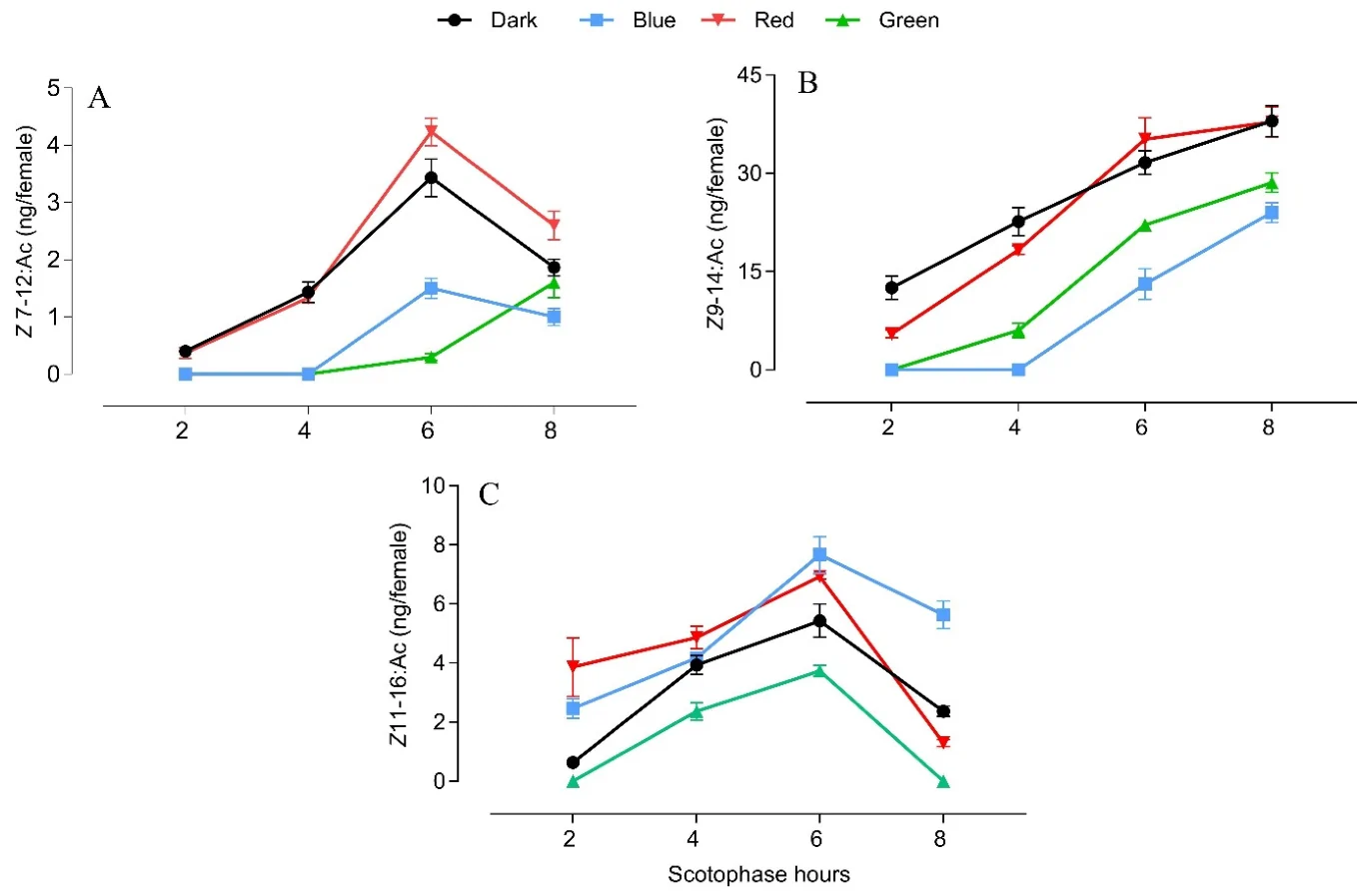
Temporal dynamics of sex pheromone production in *Spodoptera frugiperda* under wavelength-specific artificial light during the scotophase. Mean (±SE) production (ng/female) of (**A**) *Z*7-12:Ac, (**B**) *Z*9-14:Ac, and (**C**) *Z*11-16:Ac from individual pheromone glands (*n* = 3, replicates per treatment) at hours 2, 4, 6, and 8.

The temporal production of all three pheromone compounds was significantly influenced by light treatment, time, and their interaction (Figure 6). For *Z*7-12:Ac, all three effects were highly significant (light: F_(3,32)_ = 102.795; time: F_(3,32)_ = 147.281; interaction: F_(9,32)_ = 20.029, *p* < 0.001). Dark and red light supported detectable *Z*7-12:Ac production from hour 2, peaking at hour 6, whereas blue and green light completely suppressed production during the early scotophase with measurable quantities only appearing from hour 6 onwards. Across the full time course, red and dark treatments produced significantly more *Z*7-12:Ac than blue and green, which did not differ from each other (Tukey’s HSD, *p* = 0.570). For *Z*9-14:Ac, significant effects of light (F_(3,32)_ = 90.925), time (F_(3,32)_ = 221.239), and their interaction (F_(9,32)_ = 3.819, *p* = 0.002) were observed. Dark and red light supported continuous *Z*9-14:Ac accumulation from hour 2, reaching approximately 38 ng/female by hour 8, whereas blue light showed no detectable production until hour 6. Over the time course, dark and red were statistically equivalent and both significantly enhanced the production of *Z*9-14:Ac compared to blue and green (Tukey’s HSD, *p* = 0.001). For Z11-16:Ac, significant effects of light (F_(3,32)_ = 30.642), time (F_(3,32)_ = 48.968), and their interaction (F_(9,32)_ = 4.799) were detected. Unlike the other two compounds, *Z*11-16:Ac peaked at hour 6 across all treatments and declined thereafter. Under blue light, *S. frugiperda* females produced the highest amount of *Z*11-16:Ac at hour 6 and maintained significantly greater overall production than dark and green light throughout the scotophase, while blue and red did not differ significantly (Tukey’s HSD, *p* = 0.245). Green light was the only treatment in which *Z*11-16:Ac was undetectable at hour 8.

### 3.6. Influence of Artificial Lights on Mating Success in Semi-Field Conditions

**Figure 7:**
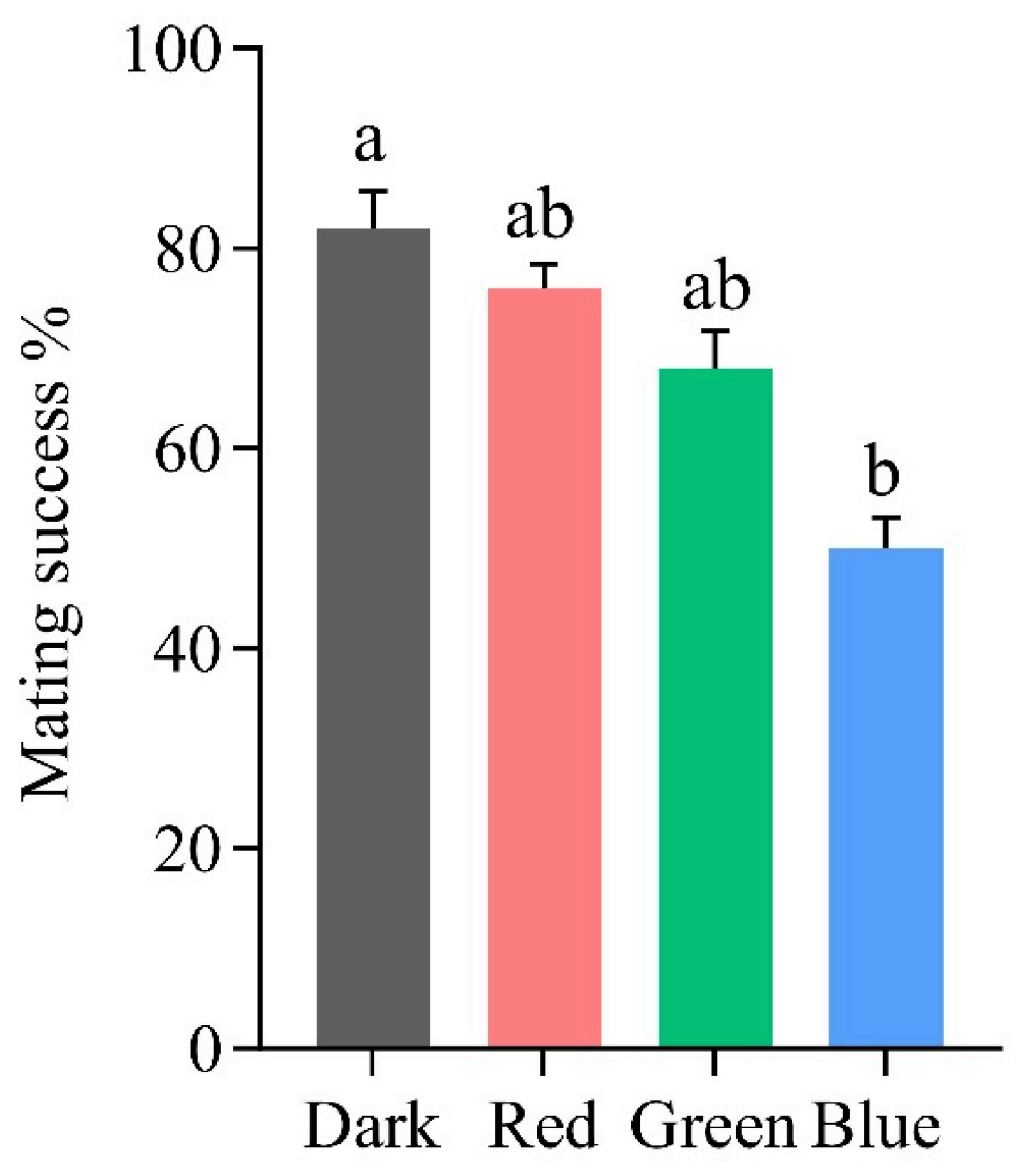
Effect of wavelength-specific artificial light on mating success of *Spodoptera frugiperda* under semi-field conditions. Mating success is expressed as the proportion of females confirmed mated by egg hatch under dark control, red, green, and blue light conditions (*n* = 5, per treatment). Bars represent mean (±SE) standard error from a generalized linear model with binomial error distribution. Different letters indicate significant differences among treatments (Bonferroni-adjusted pairwise comparisons, *p* < 0.05).

Light treatment significantly affected mating success of *S. frugiperda* under semi-field conditions (Wald χ^2^(3) = 12.844, *p* = 0.005; Figure 7). Mating proportions ranked in descending order were as follows: dark control (0.82 ± 0.05), red light (0.76 ± 0.06), green light (0.68 ± 0.06), and blue light (0.50 ± 0.07). Blue light was the only treatment to significantly reduce mating success relative to the dark control (*p* = 0.002) and red light (*p* = 0.031), representing a 39% reduction in mating probability relative to dark conditions. In contrast, red and green light treatments did not differ significantly from the dark control or from each other (*p* ≥ 0.376), indicating that longer wavelengths exerted minimal disruption of reproductive behavior under field-relevant conditions. The consistency between semi-field and laboratory mating results, where blue light similarly produced the strongest inhibitory effect, strengthens the ecological validity of these findings and suggests that the disruptive effects of short-wavelength illumination on mating behavior persist beyond controlled laboratory settings. Collectively, these results indicate that blue light is the primary wavelength responsible for disrupting mating communication in *S. frugiperda*.

## 4. Discussion

Artificial light at night (ALAN) is a key ecological stressor that influences natural behavior and physiological activities of nocturnal insects [4]. In the present study, fall armyworm was exposed to artificial light during the scotophase, specifically, green, blue and red light conditions. Among the tested lights, blue light showed the strongest disruption across calling behavior, pheromone blend composition, and mating success, followed by green light, while red light showed minimal effects compared to dark control. These results are consistent with the general photobiological principle that nocturnal insects are highly influenced by shorter wavelengths, particularly blue [8,30,34]. The significant suppression observed under blue light likely reflects greater stimulation of photoreceptors involved in nocturnal insect light perception and circadian rhythms [35,36]. Calling behavior in moths is regulated by circadian rhythms and is restricted to specific periods within the scotophase, serving to increase the coordination between female pheromone release and male responsiveness [37,38]. In the present work, calling behavior occurred during the early to mid scotophase under dark and red light conditions, whereas it was delayed under green and blue light conditions. Similar delays in reproductive and other biological activities have been observed in other nocturnal insects exposed to ALAN [14,27,39]. The observed delay under blue and green light most likely reflects interference with circadian rhythms [18], or an alteration in perception of light cues that insect rely upon to coordinate reproductive timing [40]. In contrast, the minimal effect of red light on calling behavior aligns with reported studies that moths possess reduced sensitivity to longer wavelengths [30,41], which may describe its limited potential to disrupt circadian patterns of nocturnal insects.

Beyond disrupting the calling temporal pattern, ALAN also influenced the pheromone blend and its composition in *S. frugiperda*, with relevance to chemical communication and mate recognition. The major pheromone component of *S. frugiperda*, *Z*9-14:Ac [42], was produced at higher levels under dark control and red light conditions, whereas blue light suppressed *Z*9-14:Ac production. Although *Z*7-12:Ac showed lower production under blue illumination, this difference was not statistically significant. In contrast, increased production of *Z*11-16:Ac under blue light, compared to all other treatments, may further reduce the mating success [43]. In moths, male attraction relies not only on key pheromone compounds but optimal ratios of components play a key role, and even minor variations in species-specific blends can impair mate recognition [20,23]. The light-induced changes in pheromone composition observed here suggest that ALAN may simultaneously disrupt multiple physiological pathways involved in pheromone biosynthesis and regulation [15,25] rather than suppressing pheromone production. We note, however, that these physiological pathways, circadian regulation, PBAN signaling, and pheromone biosynthesis, were not directly measured in this study; the interpretations offered here should therefore be regarded as hypotheses to be tested in future work rather than as demonstrated causes. The combined effects of delayed calling behavior and altered pheromone composition resulted in reduced mating success, specifically under blue light conditions. Successful mating in moths requires coordination between female signaling and male responsiveness behavior, and disruption in either mechanism may inhibit mating success [4,21,23,44]. A recent study further supports this interpretation, demonstrating that blue and green light reduced survival, development, and fecundity across developmental stages of fall armyworm, with the maximum reproductivity reported under red light conditions [30]. In combination, these results showed that ALAN not only disrupts behavior or chemical communication but interferes with broader reproductive mechanisms of *S. frugiperda*.

The consistency between our laboratory and semi-field experiment results strengthens the ecological validity of these findings. This suggests that light-induced mating disruption also persists under field conditions and is not limited to controlled laboratory conditions. Previous studies similarly reported ALAN effects on nocturnal insect behaviors and reproduction under field conditions [14], and positive phototaxis recorded towards green and blue light in moth species, including fall armyworm [29,34,45], emphasizes the practical relevance of these wavelengths in agroecosystems where artificial illumination is prevalent. These findings connect the applied agricultural perspective taken here with the broader conservation literature on ALAN and moths, in which nighttime lighting has been linked to disrupted moth behavior and to population-level declines across nocturnal moth communities [4,16,46,47]. Wavelength-informed lighting may therefore help identify strategies that suppress pest reproduction while limiting harm to non-target nocturnal insects. The demonstration that specific light conditions disrupt reproductive behavior at behavioral and physiological levels in fall armyworm suggests that specific alterations in artificial illumination may offer a non-chemical approach to suppress moth reproduction. Blue light, which significantly reduced mating under semi-field conditions, supports further investigation as a component of light-based pest management strategies. The present study focused on the calling behavior of females, pheromone production, and mating success; however, male responsiveness to females under light conditions was not directly quantified. Because males and females were exposed to the same light environments, the observed reduction in mating success cannot be attributed solely to altered female signaling, it may equally reflect direct effects of light on male behavior and responsiveness, or a combination of both. Separating the female and male contributions is an important priority for future work. A key limitation of this study is that the light intensities used (1005–1440 lux) are one to several orders of magnitude higher than ecologically realistic light pollution levels, which at ground level are typically below a few lux. Our results should therefore be interpreted as responses to bright, wavelength-specific nighttime light exposure of the kind encountered near light traps and LED installations in applied pest management, rather than a simulation of ambient artificial light at night. The magnitude and underlying mechanisms of these responses may differ at low, ecologically realistic intensities, and this should be tested directly in future work. Therefore, future studies examining males exposed to light conditions, and analyses of their antennal electrophysiological responses, would be helpful. Specifically, electroantennogram (EAG) or single sensillum recording (SSR) assays using synthetic pheromone compounds or light-exposed female pheromone extracts would help determine whether the observed reduction in mating reflects weakened male detection towards females or is because of altered pheromone blends, or both.

## 5. Conclusions

The present study demonstrates that wavelength-specific artificial light at night has significant effects on the pheromone-mediated reproductive communication system of fall armyworm. Blue light produced the most pronounced disruption, significantly delaying calling behavior, suppressing *Z*9-14:Ac, and elevating *Z*11-16:Ac, altering the species-specific pheromone blend and substantially reducing mating success in both laboratory and semi-field conditions. Green light produced intermediate effects under laboratory conditions, although its inhibitory effect was not significant in semi-field conditions. Red light produced comparable effects to dark control conditions across all parameters. The consistency between laboratory and semi-field results for blue light strengthens the robustness of this effect and confirms that short-wavelength light acts as a behavioral and physiological stressor for *Spodoptera frugiperda*. From an applied standpoint, blue-wavelength illumination shows potential as a non-chemical IPM component, while red light produced minimal disruption at the intensities tested. Because these intensities exceed ecologically realistic light pollution levels, the relevance of these findings to ambient nighttime lighting should be confirmed at lower intensities. Future research should quantify male antennal responses to light-exposed female extracts, assess varying light intensities and durations, and evaluate impacts under open-field conditions.

## Data Availability

The original contributions presented in this study are included in this article. Further inquiries may be directed to the corresponding author.

## Abbreviations

The following abbreviations are used in this manuscript:

ALAN	Artificial light at night
PBAN	Pheromone biosynthesis activating neuropeptide
Z9-14:Ac	(Z)-9-tetradecenyl acetate
Z7-12:Ac	(Z)-7-dodecenyl acetate
Z11-16:Ac	(Z)-11-hexadecenyl acetate
GC–MS	Gas chromatography mass spectrometry
TIC	Total ion chromatograph
EI	Electron impact
HPLC	High-performance liquid chromatography
EAG	Electroantennogram
SSR	Single sensillum recording
